# Insights into the Structure, Correlated Motions, and Electrostatic Properties of Two HIV-1 gp120 V3 Loops

**DOI:** 10.1371/journal.pone.0049925

**Published:** 2012-11-19

**Authors:** Aliana López de Victoria, Phanourios Tamamis, Chris A. Kieslich, Dimitrios Morikis

**Affiliations:** Department of Bioengineering, University of California Riverside, Riverside, California, United States of America; Weizmann Institute of Science, Israel

## Abstract

The V3 loop of the glycoprotein 120 (gp120) is a contact point for cell entry of HIV-1 leading to infection. Despite sequence variability and lack of specific structure, the highly flexible V3 loop possesses a well-defined role in recognizing and selecting cell-bound coreceptors CCR5 and CXCR4 through a mechanism of charge complementarity. We have performed two independent molecular dynamics (MD) simulations to gain insights into the dynamic character of two V3 loops with slightly different sequences, but significantly different starting crystallographic structures. We have identified highly populated trajectory-specific salt bridges between oppositely charged stem residues Arg9 and Glu25 or Asp29. The two trajectories share nearly identical correlated motions within the simulations, despite their different overall structures. High occupancy salt bridges play a key role in the major cross-correlated motions in both trajectories, and may be responsible for transient structural stability in preparation for coreceptor binding. In addition, the two V3 loops visit conformations with similarities in spatial distributions of electrostatic potentials, despite their inherent flexibility, which may play a role in coreceptor recognition. It is plausible that cooperativity between overall electrostatic potential, charged residue interactions, and correlated motions could be associated with a coreceptor selection and binding.

## Introduction

Cell entry by HIV-1 occurs through the interaction of the surface envelope glycoprotein (consisting of subunits gp41 and gp120) with their primary receptor CD4 and coreceptors CCR5 and CXCR4 on the surface of host cells [1]–[5]. Initial contact of gp120 with CD4 is followed by a structural rearrangement in gp120 that exposes the V3 loop in order to interact with CCR5 (infecting mostly macrophages) or CXCR4 (infecting mostly T-cells) [6]–[10]. Coreceptor binding induces further conformational changes in gp120 and dissociation of gp41 that facilitate attack of gp41 on the host cell membrane, thus enabling fusion of the viral and host cell membranes. Fusion results to viral entry, with end result cell infection by release of viral content.

Despite the high sequence variability and structural flexibility of the V3 loop, a mechanism exists that allows the recognition and binding with coreceptors CCR5 (R5-tropic strains) or CXCR4 (X4-tropic strains). It has been proposed that this mechanism involves charge complementarity and electrostatic interactions between the V3 loop and the N-terminal and extracellular loop 2 (ECL2) domains of CCR5 or CXCR4 [5], [11]–[21]. It has also been proposed that the switch from selecting coreceptor CCR5 to CXCR4 as the diseases progresses, involves increase of the net charge of the V3 loop [10]–[12], together with the disappearance of the glycosylation motif N6X7T8|S8X9 (where X≠Pro) [8] and the presence of positively charged residues at one or more of positions 11, 24 and 25, known as the “11/24/25” rule [9]. However, the structural origin of the interaction is not well understood. In this study we use molecular dynamics simulations to better understand the structure and dynamics of the V3 loop and how they affect its electrostatic profile. We chose to perform molecular dynamic simulations for the V3 loop alone, as opposed to whole gp120, because we expect that this work will form the basis for inhibitor design, based on V3 loop-derived peptides.

**Figure 1:**
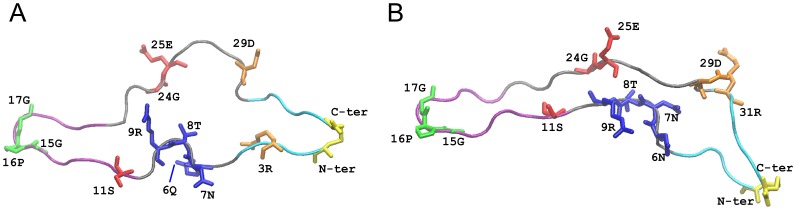
Molecular model of the V3 loops for 2B4C (A) and 2QAD (B). Backbone is shown in tube representation. The base (residues 1–4, 31–35), stem (residues 5–10, 21–30) and tip (residues 11–20) regions are colored in cyan, black and purple color, respectively. Specific side chain residues are shown and are color-coded: yellow denotes residues 1 and 35, forming the disulfide bridge; blue denotes residues involved in the glycosylation motif; red denotes residues involved in the “11/24/25” rule; green denotes the conserved GPG motif at the tip of the loop; orange denotes residues involved in salt bridges within the present MD simulations, including residue 9 (blue in 2B4C and 2QAD) and residue 25 (red in 2QAD).

The V3 loop is composed of 31–39 residues, it is positively charged, and it is maintained in a loop conformation by a disulfide bridge, linking its amino and carboxy termini (Figure 1). It consists of three distinct regions: the base (closer to the core of gp120 and including the disulfide bridge), the tip (or crown) at the opposite end from the base, and the stem between the base and the tip. The V3 loop is solvent exposed and highly dynamic. Because of its dynamic character, the V3 loop was absent in the early gp120 crystallographic structures, due to lack of resolved electron density. In two crystallographic structures, the gp120 was properly stabilized because of complex formation with CD4 and antibodies and the V3 loop was structurally resolved. The two V3 loop structures differ by three mutations only, and, interestingly, they display differences in their secondary structures [4]–[5].

Significant experimental efforts have been made to pinpoint the exact residues of CCR5 and CXCR4 that interact with gp120, but because of differences in methods and HIV strains, the results are mixed; however general conclusions are reached (reviewed in Ref. [21]). In summary, in the case of CCR5, point mutations of negatively charged residues at the N-terminus showed their involvement in binding. In addition, insertion of CCR5 N-terminus (CCR5-Nt) into other chemokine receptors resulted in chimeric receptors capable of mediating viral infection, therefore demonstrating the involvement of the N-terminus in viral activity [21]. In the case of CXCR4, mutations of negatively charged residues at the N-terminus and ECL2, or the removal of a glycosylation site within the N-terminus, resulted in weaker interactions or inhibition of viral entry [21].

Computational studies have employed the use of molecular dynamics (MD) simulations or docking methods to understand the dynamic characteristics of gp120 and its interaction with the receptor and coreceptors in the host cell [22]–[25]. The studies have suggested that gp120 undergoes conformational changes upon CD4 binding, which is in line with isothermal calorimetry studies that showed entropy loss upon binding [26]. Binding increases the overall rigidity of gp120, but retains the high level of flexibility of the V3 loop [24]–[25]. Additional computational studies suggested that the highest mobility of V3 occurs in the CD4-free states [26]. Moreover, further computational studies suggested that, along with the bridging sheet, V1/V2 stem, the V3 loop can move in concert with the CD4-complexed gp120 domains, while simultaneously participating in complicated combinations of variable motional modes such as rotation/twisting, opening/closing, or closing/flexing [27]. As a result it is suggested that these perplexing concerted motions are associated with the dynamics mechanism of gp120-receptor association/release, and also provide one possible explanation for the failure of gp120 to induce potent neutralizing antibodies against the CD4 and coreceptor binding sites [27]. In a subsequent computational study, the authors show that the V3 loop in the unliganded gp120 moves in close concert with the structural components neighboring the V3 loop base, whereas the V3 loop is an isolated rigid unit, within the framework of the CD4-complexed gp120, as it moves almost uncorrelated with other structural component [28]. Therefore, it is speculated that the association of CD4 results in both the “detachment” of the V3 loop from its adjacent components and the increased V3 loop freedom, and, in addition, the increased flexibility of the V3 in the bound-CD4 state could enable the subsequent recognition of the coreceptor CCR5 or CXCR4 [28]. A more recent hybrid MD and experimental study went further to suggest that mutations in the V3 loop that result in net charge switch from +3 to +7 can be associated with conformational changes that modulate binding to CD4, coreceptor, and antibodies, and affect the neutralization sensitivity of HIV-1 by anti-CD4 monoclonal antibodies [29].

NMR studies examining the structure of the V3 loop indicate that in an R5-tropic V3 sequence, a favorable electrostatic interaction can occur between Arg9 (numbering scheme of present study) and Glu25, which are positioned opposite to each other in a β-hairpin V3 loop conformation stabilized by an antibody. The authors of this study propose that an electrostatic interaction can stabilize the β-hairpin conformation and dictate the residue-pairing across the two β-strands [30]. Another study of three active V3 loop-derived peptides, showed by titration and NMR chemical shift perturbation and NOE experiments that the strength of the interaction between peptides and a CCR5-Nt peptide is dependent on the number of positive charged residues in the V3 loop peptides [31]. This is in agreement with previous experimental (binding and inhibition assay) and computational (electrostatic calculation) studies, which had proposed that as the V3 loop peptide positive charge increases, the interaction between the V3 loop peptides and the CCR5-Nt peptide is augmented [18]–[20]. Specifically, 2 to 5 arginines (depending on the peptide) were involved in chemical shift perturbations upon CCR5-Nt titration [31]. NMR studies of a CCR5-Nt peptide have demonstrated a short helical conformation and accompanying docking calculations have shown that the CCR5-Nt peptide shows preference for binding at the base of the V3 loop [5]. The same studies showed involvement of pairwise electrostatic interactions between Arg31 (numbering scheme of present study) of the V3 loop and a sulfated tyrosine in CCR5-Nt. Kwong and coworkers discuss the electrostatic complementarity between the V3 loop and the two sulfated tyrosines of CCR5-Nt, and suggest that binding of CCR5-Nt alters the V3 base, by forming a pocket to accommodate the two sulfated tyrosines, and induces the formation of rigid β-hairpin in the V3 loop [5].

The aforementioned studies indicate V3 loop flexibility in the free state, and stabilization in bound states, as well as the involvement of electrostatics in both structural stability and interactions with coreceptors. In the present study we have performed explicit solvent molecular dynamics simulations of the two HIV-1 V3 loops from two different crystallographic structures with intact V3 loop [4]–[5], to obtain insights into possible intramolecular stabilizing interactions and to assess if correlated motions are encountered within the MD simulations. The results reveal that, despite specific structural differences, the two V3 loops undergo common correlated motions within the trajectories, in cooperation with specific charged interactions between residues in the stems. This observation can probably be associated with the coreceptor binding specificity.

## Methods

### V3 Loop Structural Templates

Two crystal structures of gp120 were obtained from the Protein Data Bank (PDB [32]), where the V3 loop was complete, and used as initial structural templates. The PDB codes are 2B4C [4] and 2QAD [5]. In 2B4C, the gp120 with V3 isolate JR-FL was bound to CD4 and the antigen-binding fragment (Fab) of the X5 antibody. In 2QAD, gp120 with V3 isolate YU2, was in complex with CD4 and 412d, a functionally sulfated antibody. In the present study, we retained only the coordinates of the V3 loop, from both structures. Both V3 loop structures start at position 296 and end at position 331. In the case of 2B4C four amino acids have double conformations, from which conformation A was retained. In both structures residue positions 310–311 were blank, whereas position 322 was occupied by two residues; as a result, the total length of the V3 loop peptides is 35 residues. In the present study, we have renumbered the atoms and amino acids starting from position 1 and ending in position 35. The two V3 loop peptides will be referred hereafter by their crystallographic PDB code, 2B4C and 2QAD. There are three mutations when comparing the V3 loop sequences of 2B4C and 2QAD, namely N6Q, H13N, and F20L, where the residue on the left of the sequence number corresponds to 2B4C and that on the right corresponds to 2QAD.

### Molecular Dynamics Simulations

All MD simulations were conducted with the program NAMD, version 2.7 [33]. The V3 loop atomic charges, van der Waals and stereochemical parameters were taken from the CHARMM22 all-atom force field [34], including a CMAP backbone φ/ψ energy correction [35] and indole parameters [36]. The disulfide bond between the first and last cysteine of the V3 loop was fixed using the patch DISU. The V3 loops were immersed in a water box of 45×47×64 Å.

The structures were subjected to a 1000 steps of energy minimization prior to the simulations. A 100-ns simulation was performed for each V3 loop structure, using explicit solvent model, under constant number of particles, pressure, and temperature conditions (298K). The molecular dynamics time step was set to 2 ps. The system was neutralized in each simulation, and the ionic strength was set to 150 mM by addition of sodium and chloride ions. Coordinates were sampled every 10 ps, to generate 10,000 snapshots during the trajectory. Analysis of the trajectories was performed by a series of in-house scripts, using FORTRAN and R programming language (Foundation for Statistical Computing, Vienna, Austria, 2009; http://www.R-project.org), and analysis software packages Bio3D [37] and Wordom [38], in conjunction with Chimera [39] and VMD [40].

### Per-Residue Interactions

The interaction side chain free energies were computed using.
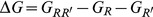
(1)where *RR’*, *R* and *R’* denote, respectively, the free energy of side chain groups *RR’*, *R* and *R’*. Individual free energies, *G*, were computed via the Molecular Mechanics-Generalized Born Surface Area (MM-GBSA) approximation [41]–[46] by removing all non-protein atoms (e.g. water molecules and ions) from the simulation trajectories and applying the equation
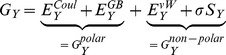
(2)where Y corresponds to RR’, R or R’. The first three terms on the right-hand side of Eq. (2) are, respectively Coulomb, Generalized-Born, and van der Waals energies and SY is the solvent-accessible surface area (SASA) of state Y. The GBMV2 generalized-Born model was used for GB term [47]–[48]. Previous studies have confirmed the high precision of the GBMV2 model in evaluating solvation energies by comparing it with accurate Poisson-Boltzmann calculations [48]. The coefficient σ was set to 15 cal/mol/Å2 as in [48].

We employed Eq. 3 to calculate the interaction energies between two side chain groups of atoms (R and R’).
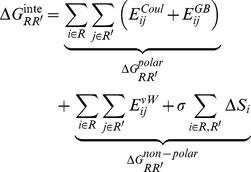
(3)


In the calculations, the side chain interaction energy of residue *R* corresponds to the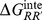, where subset *R* includes all the side chain atoms of residue *R*, while *R’* includes the side chain atoms of the entire peptide excluding the side chain atoms of residue *R*. To compute the GB term in Eq. (3), we included all the atoms of the peptide and set the charge to zero for atoms outside the groups *RR’*, *R* and *R’*, respectively, in each calculation of the terms on the right-hand side terms of Eq. (1). Backbone atoms were included in all GB energy evaluations with zero charge, aiming at including the backbone screening effect between interacting side chain atoms. The last term represents the difference in the SASA value between two distinct cases: (i) considering the two side chain groups *R*, *R’* as interacting, and (ii) considering the two side chain groups *R*, *R’* as non-interacting (e.g. by replacing R/R’ with solvent when calculating, the R’/R SASA value, respectively).

### Dynamic Cross-Correlation Analysis

We created Dynamic cross-correlation maps (DCC) to represent dynamic cross-correlated displacements of C_α_ atoms across each MD simulation run. Prior to the execution of the analysis all frames were superimposed against the initial structure of each system. The cross-correlation matrix elements for two *i* and *j* C_α_ atoms, *C_ij_*, are defined by the equation.
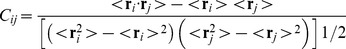
(4)


The vector **r** *_i_* denotes the position of atom C_α_^i^, and<>denotes the time average over the entire trajectory. The calculated matrix was analyzed using Wordom [38]. For completely correlated motions 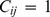, and for completely anti-correlated motions 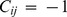. 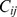 elements do not include any information about the magnitude of the motions, which can be small local oscillations as well as large scale collective motions. Complete correlation corresponds to motions with the same phase and same period. Deviations from 1 (or −1) imply either that the motions of *i* and *j* are less correlated (or anti-correlated) or that they deviate from motion along a straight line [49]–[54]. Prior to the execution of DCC analysis, all frames were superimposed.

### Principal Component Analysis

Although the DCC analysis provides an overview of the correlated motions between pairs of atoms, it does not provide a thorough picture of the complexity of collective principal atomic motions. To extract the principal motions encountered within the MD simulations from the irrelevant noise, we conducted a Principal Component Analysis (PCA) over the entire MD trajectories in each of the two systems by diagonalizing the covariance matrix *S* of the C_α_ atom position deviations with respect to the average structure. Prior to the execution of the analysis all frames were superimposed against the initial structure of each system. Matrix elements *S_ij_*, corresponding to the numerator of *C_ij_*, are defined by the following equation.
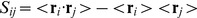
(5)


The diagonalization of matrix S aims at obtaining an orthogonal set of eigenvectors. The lowest frequencies correspond to the eigenvectors with large eigenvalues, and consequently, they represent the largest concentrated motion of protein related its function. Previous computational studies employed both DCC and PCA to elucidate the motions observed within the simulations [49]–[54]. In the present study we combine DCC and PCA by applying DCC on the motions of the principal component eigenvectors; specifically, we examine the positional variation within the principal components by creating DCC maps to study the motion of C_α_ atomic coordinates observed in each principal eigenvector. The analysis is conducted with Wordom [38] and in-house FORTRAN programs.

### Free Energy Landscapes

The trajectories explored in each system were projected on the free energy landscape (FEL), as in reference [55], using as reaction coordinates the projection of each trajectory along the first (*PC1*) and second (*PC2*) principal components. Free energy landscapes were constructed by dividing the *PC1, PC2* subspace into grids and by subsequently calculating the two-dimensional probability *P(PC1,PC2)* as.
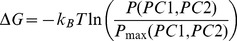
(6)where *P*_max_ *(PC1,PC2)* corresponds to the grid with the maximum probability of occurrence in each system. This process aimed at deriving the global and local free energy minima corresponding to distinct representative conformations encountered in each of the two systems during the MD simulations. To extract the most representative structure for every minimum in both systems, a cluster analysis employing all C_α_ atoms was conducted via Wordom [38], using snapshots of every minimum separately and a clustering radius of 2 Å. The representative structures of the global minimum and the local minima were extracted from the cluster centers of the most populated cluster in each minimum-basin of the FELs.

### Electrostatic Calculations

Clustering of electrostatic potentials depicts electrostatic similarities of members of protein families, which can be correlated to biological properties and functions. In the present study, we calculated electrostatic potentials and performed hierarchical clustering for 200 snapshots, 100 from each of the two V3 loop MD trajectories. The two parent structures were initially superimposed against each other and additionally each of the trajectories were superimposed on their respective parent structures to allow comparison across the two different trajectories. Poisson-Boltzmann electrostatic calculations and hierarchical clustering analysis were performed as previously described using the computational framework AESOP (Analysis of Electrostatic Similarities Of Proteins [56]–[60]). The program PDB2PQR [61] was used to prepare the V3 loop coordinates for electrostatic calculations by including van der Waals radii and partial charges for all atoms according to the PARSE force field [62]. Electrostatic potentials were calculated using the program APBS (Adaptive Poisson Boltzmann Solver [63]) and the linearized form of the Poisson-Boltzmann equation. A box with 129×129×129 grid points was used. The box dimension for the V3 loop snapshots were 65 Å×65 Å×65 Å. The molecular surface was calculated using a probe sphere with a radius of 1.4 Å, representing the radius of a water molecule. The dielectric coefficients were set to 2 and 78.54 for the protein interior and solvent, respectively. The ion accessibility surface was calculated using a probe sphere with a radius of 2.0 Å, representing monovalent counterions. Calculations were repeated with ionic strengths corresponding to 0 mM salt concentration (representing Coulombic interactions within the protein unscreened by solvent counterions and implicitly approximating the effects of dynamics [57]) and 150 mM (representing physiological ionic strength in serum).

Electrostatic similarity distances (ESDs) were calculated according to.
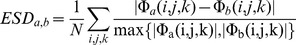
(7)where Φ_ a_ and Φ_b_ are the electrostatic potentials of proteins a and b at grid point (i, j, k) and N is the total number of grid points. This error-type relation compares the spatial distributions of electrostatic potentials of pairs of proteins. The normalization factor of the denominator assures small values in the 0–2 range, with 0 corresponding to identical spatial distributions of electrostatic potentials and 2 corresponding to totally different spatial distributions of electrostatic potentials. Classical multidimensional scaling was performed using the cmdscale function of R programming language. Multidimensional scaling converts a matrix of dissimilarities (ESD distances in our case) into a dimensional representation of a set of points, such that the distances between points are approximately equal to the dissimilarities.

## Results

### RMSD/RMSF

Root mean square deviation (RMSD) and root mean square fluctuation (RMSF) for each V3 loop structure are shown in Figure S1. Both structures are very flexible throughout the entire trajectories as depicted from the high RMSD values in the range of 4–9 Å and possess similar RMSF profiles. The RMSF profiles reveal that the GPG region, a highly conserved motif in the V3 loop, is the most highly fluctuating moiety; this motif is found at the tip of the loop (the most distant part from the gp120 core) and as a result, it is expected to be highly flexible in the context of the entire gp120 protein, as well. In addition, the disulfide bridge and the residues proximal in sequence to the disulfide bridge are highly flexible in both systems. The 2B4C is somewhat more flexible compared to 2QAD due to slightly larger fluctuations observed within the 24–29 residue loop-like moiety in the former compared to the 24–29 residue linear-like moiety in the latter (Figure 1).

**Table 1:** Occupancy of most prevalent hydrogen bonds, involving side chains and/or backbone, throughout the trajectories.

Structure: 2B4C							Structure: 2QAD						
Residue #1			Residue #2			%	Residue #1			Residue #2			%
ARG	9	NH2	ASP	29	OD1	68.1^a^	ARG	31	NH2	ASP	29	OD2	74.6^a^
ARG	9	NH2	ASP	29	OD2	65.2^a^	ARG	31	NH2	ASP	29	OD1	73.6^a^
**HIS**	**34**	**N**	**THR**	**2**	**O**	**58.6**	ILE	27	N	ASN	7	O	70.2
**THR**	**2**	**N**	**HIS**	**34**	**O**	**57.3**	ARG	9	N	GLU	25	O	68.5
ASN	7	N	ASN	5	OD1	52.4	ARG	3	NH1	CYS	35	OXT	48.4
ARG	9	NH1	ASP	29	OD2	50.1	ARG	3	NE	CYS	35	O	47.5
SER	11	N	GLN	6	O	47.1	ARG	31	NE	ASP	29	OD1	45.2
ARG	9	NH1	ASP	29	OD1	44.1	ARG	31	NE	ASP	29	OD2	42.3
ARG	9	N	ASN	5	O	41.8	ALA	33	N	ASP	29	O	41.0
LYS	10	N	GLN	6	O	40.4	ARG	31	N	ASP	29	OD1	39.3
ARG	31	N	GLY	28	O	39.8	ARG	31	N	ASP	29	OD2	37.0
GLN	32	N	GLY	28	O	35.4	CYS	35	N	GLN	32	O	34.8
**GLN**	**32**	**N**	**ASP**	**29**	**O**	**32.3**	**THR**	**2**	**N**	**HIS**	**34**	**O**	**32.8**
LYS	10	N	ASN	7	O	32.1	**ARG**	**18**	**N**	**GLY**	**15**	**O**	**32.7** b
THR	8	N	ASN	5	OD1	27.8	**GLN**	**32**	**N**	**ASP**	**29**	**O**	**32.5**
ARG	9	NE	SER	11	OG	26.0	ARG	3	NH1	CYS	35	O	32.3
GLN	32	NE2	GLY	28	O	25.6	ARG	9	NH1	GLU	25	OE1	31.2^a^
**ARG**	**18**	**N**	**GLY**	**15**	**O**	**23.8** b	**HIS**	**34**	**N**	**THR**	**2**	**O**	**30.6**
ARG	3	NH1	GLN	32	O	22.4	GLY	24	N	THR	22	OG1	30.4
ASN	7	ND2	ASN	5	OD1	21.8	ARG	9	NE	SER	11	OG	28.3
GLU	25	N	THR	23	OG1	21.4	ARG	9	NH1	GLU	25	OE2	27.1^a^
							HIS	34	N	ILE	30	O	25.8
							GLY	28	N	ASN	7	O	23.2
							ILE	27	N	ARG	9	O	22.8
							THR	8	N	ASN	6	OD1	22.7
							GLY	15	N	ARG	18	O	21.6
							SER	11	N	GLU	25	O	21.4

a Salt bridges (see Table 2).

b Hydrogen bonds present in the turn region (at the tip off each structure).

Bold font highlights bonds common in both structures. Hydrogen bonds were calculated using Chimera [39].

### Secondary Structure and Hydrogen Bonds

The initial crystallographic secondary structure profiles of the two available gp120 crystal structures with intact V3 loop are partly different (Figure 1): 2B4C possesses a short β-hairpin around the tip, while 2QAD possesses a β-hairpin with longer beta strands, extending from residue Thr8 to Ile26 and consequently the 2QAD conformation is thinner than 2B4C. A 2–34 β-bridge is present in both structures owing to the two successive hydrogen bonds among His2Ν–Thr34 O and Thr34 N – His2 O. The secondary structure was determined using STRIDE [70] implemented in VMD, and is shown in Figure S2.

Within the MD simulations, after approximately the first 12 ns, extended β-hairpins are either eliminated in 2B4C or reduced to the extent of single β-bridge among residues Thr8– Ile26 (hydrogen bonds among atoms Ile27 N – Asn7 O and Arg9 N – Glu25 O) or Lys10– Ile26 in 2QAD (hydrogen bonds among atoms Ile27 N – Arg9 O and Ser11 N – Glu25 O). The reduction of β-sheet content within the residue moiety 8–26 can possibly be attributed to the deletion of the rest of gp120 protein residues in conjunction with the high peptide flexibility. The initial 15–18 β-turn of the tip is entirely reproduced throughout both simulations, whereas consecutive 19–22 and 23–26 β-turns are mainly observed in 2B4C. β-turns within the stem residue moieties 4–10 and 27–32 are reproduced in both systems; both β-turn moieties in 2B4C and the latter moiety in 2QAD experience frequent interchanges with helical elements.

Table 1 presents the hydrogen bonds with high occupancy, for both V3 loops, throughout the trajectories. The hydrogen bonding interactions constitute basic structural elements for the β-sheet conformations and stabilize some of the β-turns encountered during the simulations.

**Table 2:** Occupancy of salt bridges throughout the trajectories. Salt bridges were calculated with a cutoff of 5 Å.

Structure: 2B4C							Structure: 2QAD						
Residue #1			Residue #2			%	Residue #1			Residue #2			%
ARG	9	CZ	ASP	29	CG	85.6	ARG	31	CZ	ASP	29	CG	91.0
ARG	3	CZ	ASP	29	CG	22.6	ARG	9	CZ	GLU	25	CD	57.5

### Salt Bridges

Persistent salt bridges occur during the MD trajectories (Table 2). In 2B4C, a very stable salt bridge occurs between Arg9-Asp29 for 85.6% of the trajectory, located between opposite stems of the loop. An additional salt bridge between Arg3-Asp29 occurs 22.6% of the time and is located at the base of the loop. These results show the salt bridge partner of Asp29 changes through the trajectory, however only 7.9% of the time both bonds occur simultaneously, making it a bifurcated bond, compared with 88.5% of the time only one salt bridge is present.

**Figure 2:**
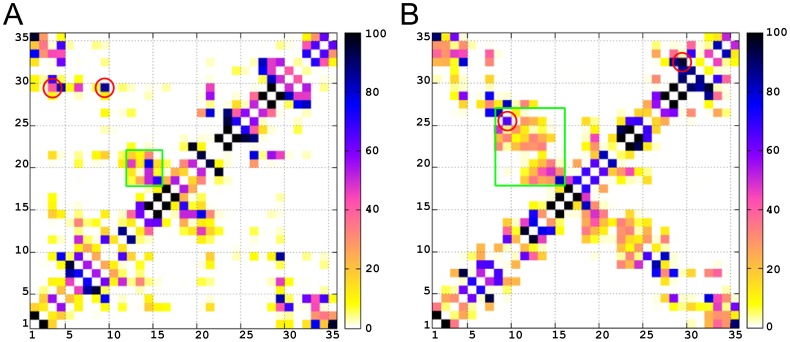
Side Chain Contact Maps for 2B4C (A) and 2QAD (B). The color code for percent occupancy is shown at the right of each figure, with 0% occupancy shown as white and 100% occupancy shown as black. Axes denote the residue number in sequence. The red circles represent the residues involved in the salt bridges. The green boxes enclose the residues involved in β-sheets.

In 2QAD, two salt bridges are observed: Arg31-Asp29 for 91% of the time and Arg9-Glu25 for 57.5%. The first salt bridge is present at the base of the loop while the second is present between opposite stems. The salt bridge involving Arg9-Glu25 is located within the beta strand of 2QAD. Occupancy of more than one salt bridge at a time was calculated; the results indicate that in 30.3% of the time only one salt bridge is present, compared to 59.1% of the time where both salt bridges are present.

Interestingly, we observe that system-specific salt bridges linking the two opposite stem sites exist in both systems, Arg9-Asp29 and Arg9-Glu25 residue pairs, in 2B4C and 2QAD, respectively. The specificity for the Arg9 partner could be attributed to the conformational selectivity which is caused by mutations. A characteristic pattern featuring positive charge in position 9 in combination with negative charge in position 25 and 29 is observed with relatively high propensity in V3 loops recognizing CCR5 coreceptors [12]. In addition, Rosen et al. demonstrate that Arg9 and Glu25 are close in proximity and facilitate the β-sheet formation [30]. Therefore, the charged residue interactions, especially those linking opposite stem regions, most likely constitute a key contributing factor both in the stabilization of conformations and the motions of the V3 loop. In addition, system-specific salt bridge between opposite stem sides may be involved in coreceptor selectivity, and, as a result, a more detailed examination of these interactions is provided below in conjunction with the dynamic cross-correlated and principal component analyses.

### Side Chain Contacts and Interaction Energies

**Figure 3:**
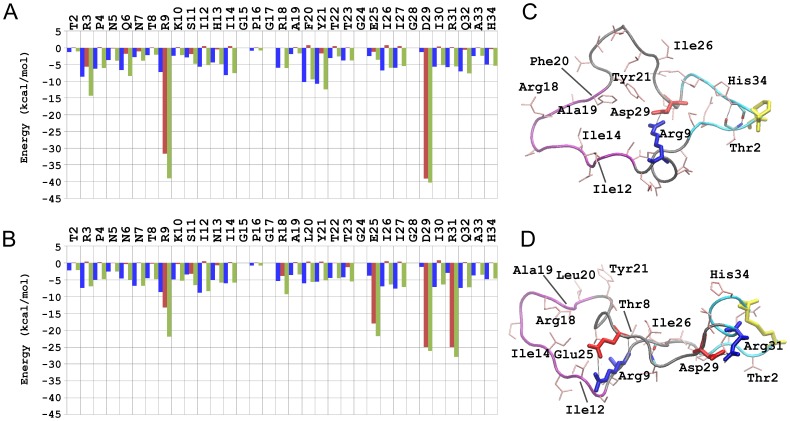
Side Chain Interaction Energies (in kcal/mol) for 2B4C (A) and 2QAD (B). Energies were computed and averaged over the trajectory, as described in Methods. The blue, red, and green bars correspond to non-polar, polar and total interaction energies, respectively. The most representative structures throughout the trajectories (global minimum; black circle in Figure 7, below) are shown in panel (C) for 2B4C and in panel (D) for 2QAD. The backbone is shown in tube representation and side chains are shown in stick representation. Negatively and positively charged residues involved in salt bridges are shown in red and blue, respectively, and disulfide bridge residues in yellow. Salt bridges and β-bridges are marked with dashed lines. The backbone of the base (residues 1–4, 31–35), stem (residues 5–10, 21–30) and tip (residues 11–20) regions is colored in cyan, black and purple color, respectively. The rest of the side chains are shown in thin pink licorice representation. Hydrogen atoms are omitted for clarity.

Side chain contact occupancy maps were calculated and plotted for each of the two systems (Figure 2). In addition, interaction energies between side chains were calculated for each of the two systems (Figure 3). Cysteine side chains (residues 1 and 35) were not considered in the interaction energy calculations, since they are bonded with the disulfide bridge. A knowledge-based combination of the side chain contact occupancies and side chain interaction energies provides insights on the role of side chain interactions in the stabilization of specific conformers throughout the MD trajectories.

The side chain contact occupancy profiles of the two systems (trajectories) are generally similar, especially in regions of mutually existing β-turns and transient helices. Owing to the closed and thinner shape of the loop in 2QAD, side chain interactions among residue moieties 4–14 and 22–28 exist almost entirely in 2QAD, and consequently, the sum of the non-polar interaction residue energies is somewhat higher in 2QAD compared to 2B4C (Figures 2 and 3).

In 2B4C, the strongest (polar) interaction is between Arg9-Asp29 (salt bridge, Tables 1 and 2, Figure 3A). The alternative salt bridge partner of Asp29, Arg3, is also highly interacting in terms of polar energy, in line with the presence of the salt bridge between Arg3-Asp29. Phe20 is located in the turn region, with its side chain pointing towards the interior of the loop, interacting strongly with a cluster of mostly non-polar residues (Ile14, Arg18, Ala19 and Tyr21; Figure 3C). These interactions, in conjunction with a hydrogen bond between Gly15-Arg18 and π-cation interaction between Arg18-Phe20, aid the stabilization of the extra β-turn in 2B4C, not observed in 2QAD due to the presence of a less bulky and non-aromatic Leu20 residue (Figure 3D). The position and interactions of Phe20 in 2B4C are considered to be responsible for the wider pattern and consecutive β-turns within the residue moiety 19–26. This result provides evidence for the existence of distinct conformational patterns between the two V3 loops and determines the specificity for the presence of two different salt bridge patterns in the two systems (e.g. Arg9 is not proximal to Glu25 in 2B4C but is proximal to Asp29).

In 2QAD, the most highly (polar) interacting residues are Arg9, Arg25, Arg29, and Arg31 mainly due to the presence of salt bridges for the pairs Asp29-Arg31 and Arg9-Glu25 (Figure 3B). The thinner shape of the loop, in combination with the presence of β-bridges in the ranges 8–26 or 10–26, is related to a rich network of polar and non-polar interactions between the residue moieties 4–14 and 22–28; these interactions are stronger in 2QAD compared to 2B4C, because 2B4C has overall wider shape in the same region.

### Dynamic Cross-Correlation

**Figure 4:**
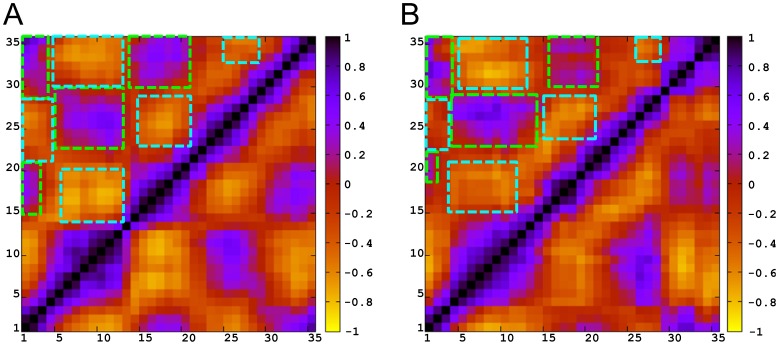
Dynamic Cross-Correlation Maps for 2B4C (A) and 2QAD (B), using C_α_ atoms. The color code for correlation or anti-correlation is shown at the right of each figure, with black being correlated, and yellow being anti-correlated. Axes denote the residue number in sequence. Green boxes denote correlated motions and cyan boxes denote anti-correlated motions.

We performed a dynamic cross-correlation (DCC) analysis on the C_α_ atomic positions throughout each of the two independent trajectories in order to examine the presence of correlated motions within the backbone of the loop. Figure 4 shows the DCC results for the two different V3 loop structures. Surprisingly, the correlated motions detected in the two systems are quite similar despite the presence of specific conformational differences between the two. Specifically, apart from the *a priori* expected correlated motions among nearby residues in sequence (along the diagonal of Figure 4), in both systems, quite specific residue segments participate in the following motions: (i) correlated motion between the two sides of the stem, incorporating approximately segments of residues 4–14 and 22–29; (ii) correlated motion between segments of residues 15–19 of the tip and segments of residues 1–3 and 33–35 of the base; and (iii) correlated motion between the two sides of the base, incorporating segments of residues 1–3 and 33–35, which are not nearby in terms of sequence but are neighboring in terms of tertiary structure (brought together by the disulfide bridge). Furthermore, (iv) less correlated and mainly anti-correlated motions are observed between approximately segments of residues 4–14 and 22–29 (stem) and segments of residues 15–19 (tip), and 1–3 and 33–35 (base). Taking into account the geometry of the closed loop, the two independent correlated motions (i) and (ii) are expected to be in a way anti-correlated between each other, as indicated by (iv). Additional insights on the anti-correlation between two correlated motions are presented in the first principal motion in the PCA.

### Principal Component Analysis

Principal component analysis (PCA) was performed using the C_α_ atomic coordinates throughout each of the two independent trajectories. The analysis aimed at capturing the motional complexity of the systems, and at identifying principal eigenvectors/motions through the elimination of irrelevant noise from motions with high frequency and less probability. To evaluate in more detail the results obtained in the PCA for each principal eigenvector/motion separately, we performed DCC analysis. We interpreted the data by analyzing molecular graphics images and DCC maps, both describing the correlated motions depicted throughout the two extreme backbone conformations.

**Figure 5:**
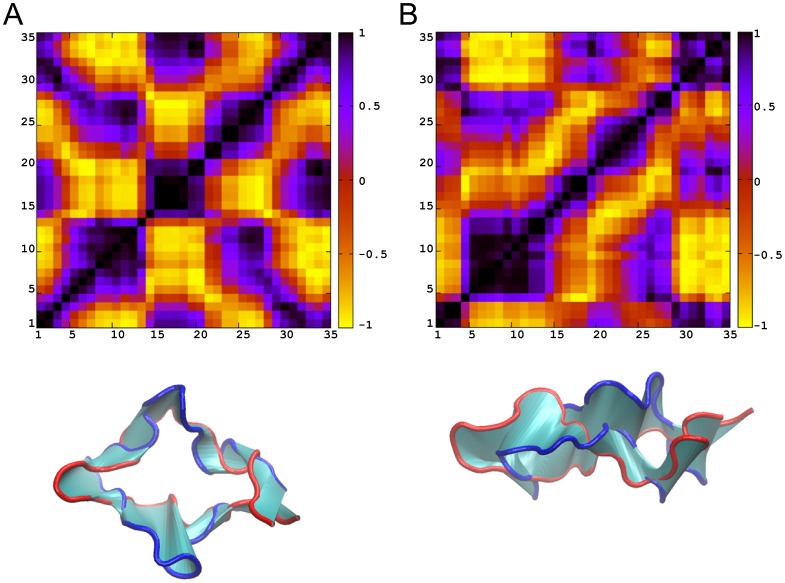
Principal Component 1 Dynamic Cross-Correlation Maps for 2B4C (A) and 2QAD (B), using C_α_ atoms. The color code for correlation or anti-correlation is shown at the right of each figure, with black being correlated, and yellow being anti-correlated. Axes denote the residue number in sequence. The bottom panels depict “extreme” structures observed during the principal components (shown in ribbon representation in blue and red) and the movements between structures (cyan). The V3 loop structures are oriented according to the residue numbering of the DCC maps, with the tip on the left and base on the right.

**Figure 6:**
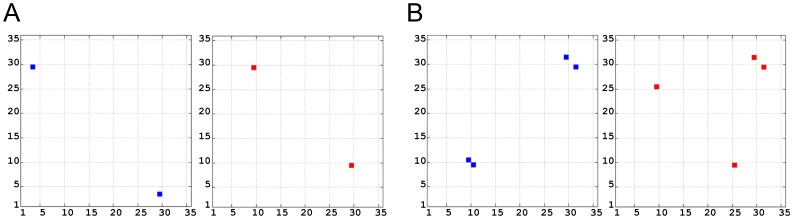
Charged Interactions within Principal Component 1 for 2B4C (A) and 2QAD (B). Axes denote the residue number in sequence. Colors correspond to those of the “extreme” structures observed during the principal component 1 of Figure 5.

In both systems, the first principal motion, which corresponds to the first eigenvector accounts for approximately half of the motions observed in each system separately (54.7% in 2B4C and 48.5% in 2QAD). The correlated motions, depicted from the DCC maps of the first principal motions (Figure 5), are almost identical and are in line with the DCC maps created for the entire trajectories in the two systems (Figure 4). As expected, the irrelevant noise from other motions is discarded and the results provide a clear overview of the major motion in both systems. According to the results, in the major motion, as the two stem sides (approximately residues 4–14 and 22–29) approach each other (from blue to red color in molecular graphics of Figure 5) the tip residues (approximately 15–19) come apart from base residues (approximately 1–3 and 33–35), and vice versa, (Figure 5, bottom). It should be noted that the salt bridge-like side chain interactions contribute to the similarity between correlated motions of the two systems, since, as the two stems approach in both systems, the key and system-specific Arg9-Asp29 and Arg9-Glu25 salt bridges between opposite sides of the stem are formed in 2B4C and 2QAD, respectively (Figure 6). In addition, in 2B4C, the Arg3-Asp29 salt bridge is formed when the stem regions are apart from each other, whereas the Asp29-Arg31 salt bridge in 2QAD is maintained during the motion. Moreover, a twist of the backbone is encountered in 2QAD as the two stem sides approach each other (Figure 5).

The second principal motions of the two systems account for less than 20% in both systems, and are somewhat similar to the correlated motions of first principal motions in both systems. The two stem regions in 2QAD, or mainly stem and to a lesser extent the tip region in 2B4C, approach each other, the tip residues come apart from base residues, and vice versa (Figure S3). The main difference between the second and first principal motions in both systems is that the correlated motions concern different parts of the stems. Furthermore, the second principal motions in 2B4C and 2QAD are different with regard to their DCC motions. These variances arise due to the fact that different parts of the stems contribute to the motions in each system, and because of the system-specific organization of salt bridge-like side chain interactions (Figure S4). As in the first principal motion of 2QAD, a twist of the backbone is encountered in the second principal motion of 2QAD when the two stem regions approach each other (Figure S3).

The third principal motions of the two systems account for less than approximately 10% in both systems. The DCC motions of the third most principal motions in both systems are mutually similar and generally possess an opposite character compared to the correlated motions observed in the first principal component. In other words, as the two stem regions approach each other, the tip and base also approach each other, and vice versa (Figure S5). In contrast to the first principal motion, apart from the destruction of the Arg9-Asp29 salt bridge-like side chain interaction in 2B4C as the stems come apart, there is no apparent association between side chain charged interactions and correlated motions in the third principal motion (Figure S6). As in the two first principal motions of 2QAD, a twist of the backbone is encountered in the third principal motion of 2QAD as the two stem regions approach each other (bottom of Figure S5).

To obtain additional insights on the role of charged side chain contribution on the motions, a subsequent PCA including additionally the carbon atoms of all charged residues was applied; the results of the second PCA were almost identical the first PCA with regard to the backbone motions. Nevertheless, the inclusion of side chains enabled us to additionally trace the salt bridge-like side chain interactions among charged residues in the principal motions, described above.

### Free Energy Landscapes

**Figure 7:**
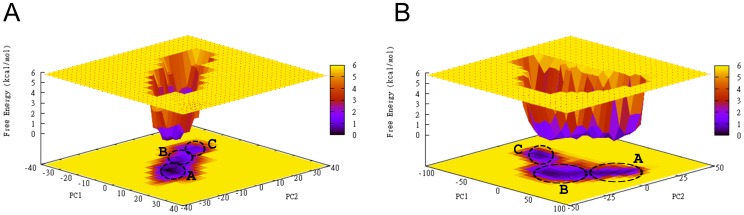
The trajectories explored in each system are projected on the free energy landscapes (FEL) using as reaction coordinates the projection of each trajectory along the first (PC1) and second PC2 principal components for 2B4C. (A) and 2QAD (B). The black circles show the free energy minima basins in the landscape, corresponding to the most representative structures throughout the trajectories; the structures are presented in Figure 3 (global minimum) and Figure S7 (second and third local minima). The first (global), second, and third free energy minima are marked with A, B, C. The color code corresponding to the free energy value (z axis in kcal/mol) is shown at the right of each panel.

We used the results of the PCA to create free energy landscapes (FELs) aiming at extracting the conformational families observed in each system. The FELs use as reaction coordinates the projection of Cα atomic positions in the simulations along the first and second principal components (see Methods) as they encompass approximately 2/3 of the simulation information. Consequently, the analysis provides additional insights into the different conformations contained within the different basins of the FELs, shown in Figure 7. In each system, three distinct basins are observed in their respective FEL, corresponding to three distinct free energy minima Figure 7.

In the 2B4C FEL (Figure 7A), the structures of the first (global) and second free energy minima basins are somewhat similar; the RMSD between the two most representative structures of the two basins is 3.6 Å. The structures within these two basins share common β-turn residue moieties (e.g. 5–10, 15–26, 28–32) and they have high populations of the Arg9-Asp29 salt bridge. The Thr2-His34 β-bridge is highly populated in the first basin structures (Figure 3C), whereas its occupancy in the second is approximately 50% (Figure S4A); a small population of 29–31 3_10_ helix is present as well in both basins. On the contrary, the structures of the third basin, despite having similar β-turn and helical conformational characteristics with regard to the first two basins, they possess an Arg3-Asp29 salt bridge and entirely lack the Arg9-Asp29 salt bridge and the Thr2-His34 β-bridge (Figure S6A).

In the 2QAD FEL (Figure 7C), the structures of the first (global) and second free energy minima basins share common characteristics in that they are stabilized by the Arg9-Glu25 salt bridge in combination with a Thr8-Ile26 β-bridge; it is worth noting that the salt bridge is more populated in the structures of the first compared to the second basin, observed approximately 50% of the snapshots of the latter. Additional differences between the structures of the two basins include (i) the presence of an additional high occupancy Thr2-His34 β-bridge only in the second basin (Figure S4B), and (ii) the presence of high occupancy α and 3_10_ helical elements in the 30–34 residue moiety of the first basin (Figure 3D). The structures of the third basin differ from the first and second basin with respect to the absence of the Arg9-Glu25 salt bridge combined with the absence of the Thr8-Ile26 β-bridge and the presence of the Lys10-Ile26 β-bridge (Figure S6B). The Asp29-Arg31 salt bridge is present with high occupancy in all basins.

A visual inspection and comparison among the structures of the two first basins, which contain the key salt bridges between opposite stem sides in both systems, and the structures of the third basin, which lack the key salt bridges between opposite stem sides in both systems, shows that the first two basins contain structures with a minimized distance between two stem sides. This proximity of the two stem sides also results in a backbone twist, in 2QAD, and a maximized tip-base distance. On the contrary, the structures of both systems in the third basin are characterized by a maximized distance between two stem regions and a minimized tip-base distance in comparison to the first two basins. These findings validate our previous results showing that the approach of stem regions is accompanied by the presence of system-specific salt bridges between residues in opposite stems. Thus, the analysis of conformations in the basins of the FELs resulted in the identification of the “extreme” structures observed in the major correlated motion in both systems. Transitions between the two extreme structures, as depicted from the major correlated motion in both systems, occur via transitions between basins A/B and basin C in the FELs (Figure 7).

### Electrostatic Clustering of Snapshots

**Figure 8:**
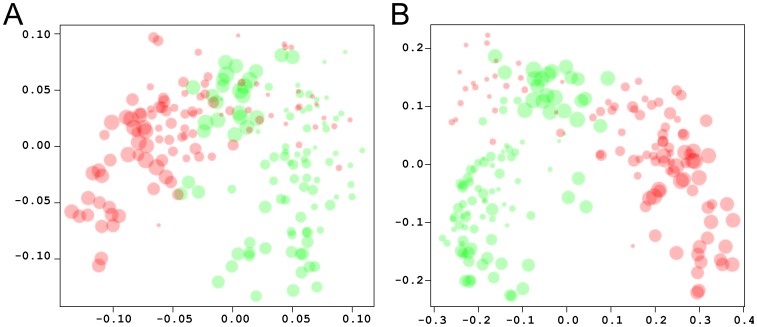
Electrostatic clustering of snapshots of the MD trajectories of the V3 loop. Electrostatic potentials were calculated using ionic strength corresponding to 0 mM (A) and 150 mM (B). The graph was generated using multidimensional scaling. The axes denote coordinates that reflect the dissimilarities between snapshots. The snapshots are defined by the diameter of the circle, with smaller circles corresponding to snapshots from the beginning of the trajectory, and gradually increasing the diameter towards snapshots from the end of the trajectory. The red circles correspond to the trajectory of 2B4C and the green circles correspond to the trajectory of 2QAD.

In addition to local and pairwise electrostatic interaction analysis, discussed above, we also performed electrostatic potential analysis to delineate global electrostatic similarities/differences between the two systems. Electrostatic calculations and hierarchical clustering analysis was performed using 100 snapshots from each trajectory, one snapshot per nanosecond. Figure 8 shows the plots that cluster the spatial distributions of electrostatic potentials of the combined 200 snapshots, calculated using 0 mM (Figure 8A) and 150 mM (Figure 8B) ionic strength. Some overlap between the electrostatic potential of snapshots is observed at both ionic strengths. For example, in Figure 8A snapshots from the beginning of the 2B4C simulation (red in Figure 8A, around 5–30 ns) are found together with snapshots towards the beginning and end of the 2QAD simulation (green in Figure 8A, 18–36 ns and 86–100 ns). A similar trend is observed in Figure 8B. The overlap of snapshots depicts instances in which both V3 loops have very similar electrostatic potentials. Despite structural variability, transient similarity of electrostatic potentials during the MD trajectories reaffirms the notion that electrostatics may play a common role in the interaction between different V3 loop sequences and coreceptors.

**Figure 9:**
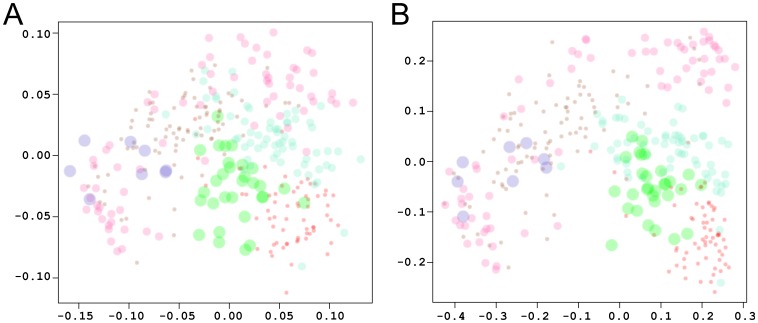
Electrostatic clustering of snapshots from the minima of the V3 loop trajectories. Electrostatic potentials were calculated using ionic strength corresponding to 0 mM (A) and 150 mM (B). The graph was generated using multidimensional scaling. The axes denote coordinates that reflect the dissimilarities between snapshots. The snapshots are defined by the diameter of the circle, with smaller circles corresponding to snapshots from the beginning of the trajectory, and gradually increasing the diameter towards snapshots from the end of the trajectory. The brown, pink, and purple circles correspond to the first, second, and third minima of 2B4C (basins A, B, C in Figure 7A, respectively). The red, teal, and green circles correspond to the first, second, and third minima of 2QAD (basins A, B, C in Figure 7B, respectively).

Similar analysis was performed using the FEL minima of each trajectory. The most representative member-structures of clusters for each minimum were selected using a clustering radius of 1.7 Å for all C_α_ atoms. (A total of 164 structures for 2B4C and 158 for 2QAD were selected.) Figure 9 shows the plots that cluster the spatial distributions of electrostatic potentials of the combined snapshots, calculated using 0 mM (Figure 9A) and 150 mM (Figure 9B) ionic strength. Some overlap between the electrostatic potential of snapshots is observed at both ionic strengths (Figure 9). This is in line with the observation of transitions between minima during the simulations, within each trajectory. There is also overlap across trajectories; for example, between the first and second minima of 2B4C and the second minimum of 2QAD (at 0 mM, Figure 9A), and between the first minimum of 2B4C and the second minimum of 2QAD (at 150 mM, Figure 9B). Interestingly, the minima of the two systems have something in common: the presence of salt bridges between opposite stems and as the two sides of the stems are close to each other the tip-base distance is maximized (in both systems).

## Discussion

The HIV-1 gp120 V3 loop is known to be highly variable in terms of sequence and structure, and for many years its structure was elusive. Determination of different structures of intact V3 loops by X-ray crystallography were made possible only through stabilization of gp120 in complexes with receptor CD4 and neutralizing antibodies [4]–[5], and provide initial conditions for atomic-level computational studies. Yet, within sequence and structural variability, some specific structural and physicochemical characteristics must be preserved that are responsible for recognition and binding to coreceptors CCR5/CXCR4 and for coreceptor selectivity (tropism), from CCR5 to CXCR4, as disease progresses.

Previous computational studies have suggested that the V3 loop intrinsic motions are uncorrelated with other structural components within the framework of the stabilized CD4-complexed gp120 [28]. This rationalizes our choice to exclude the rest of the protein, and study the structure and the motions of the V3 loop alone, in our effort to provide insights on its dynamic conformational and physicochemical profile and to form correlations with coreceptor recognition, binding, and tropism. In addition, our study aims in providing the structural basis for the design of HIV-1 entry peptidic inhibitors derived from the V3 loop, therefore a detailed analysis of V3 loop structure, dynamics, and electrostatics is necessary. The present MD simulations provide insights into the structure and the correlated motions of two V3 loops with slightly different sequences and significantly different initial crystallographic structures [4]–[5]. In line with previous experimental and computational studies [2]–[5], [13], [24]–[25] the V3 loops are highly flexible in both simulated systems.

Our MD data demonstrate that despite conformational differences throughout the trajectories of the two V3 loops, two of their major correlated motions (PC1 and PC3, accounting for about 70% of all motions) are nearly identical. These motions are coupled to the formation of specific, but distinct within the two V3 loops, salt bridge-like interactions between charged residues located in the opposite sides of the V3 loop stem. These salt bridge-like interactions have high occupancies within the MD trajectories, and are major characteristics of the lowest-energy minima in the free energy landscapes of both V3 loops. Additional transient conformational similarities at the backbone level are also present, owed to V3 loop stretching, contracting, and twisting, caused by the formation and deformation of the salt bridge-like interactions, during hoping among the lowest-energy minima of the free energy landscapes. Conformational differences are present at the side chain level, owed to variable backbones and sequence differences between the two V3 loops studied.

Previous structural NMR studies [30] depicted that a favorable electrostatic interaction can occur between Arg9 and Glu25, in CCR5-recognizing peptide, which could correlate with β-hairpin formation. Interestingly, in the present study such an interaction is reproduced in 2QAD with a simultaneous presence of a β-hairpin (isolated β-bridge) among neighboring Arg9 and Glu25 residues, specifically Thr8-Ile26 or Lys10-Ile26. In 2B4C the presence of a phenylalanine residue in position 20, pointing towards the interior of the peptide and interacting with several residues, differentiates the conformation of the peptide within the 19–26 residue moiety which adopts a loop-like local structure; as a result the structural change facilitates Arg9 to form a salt bridge with residue Asp29, probably conflicting with the formation of a β-hairpin as in 2QAD.

The role of structure [30] and motions [27]–[28] in the biological activity of the V3 loop has been suggested to be critical in terms of coreceptor recognition and tropism and in terms of penetration into the binding pocket. Furthermore, the presence of complicated combinations of variable motional modes such as rotation/twisting, opening/closing, or closing/flexing, involving the V3 loop and other gp120 fragments was suggested in previous computational studies [27]; nevertheless, to the best of our knowledge, a detailed inspection of the V3 loop intrinsic motions has not been performed yet. Our results demonstrate that despite the presence of some conformational differences between the two V3 loops investigated, they interestingly share nearly identical correlated motions, and they both contain system-specific high occupancy salt bridges between charged residues in the two opposite sides of the stem.

Another critical aspect of the V3 loop function is that it switches coreceptor selection as the disease progresses. In the early stages of infection HIV-1 selects coreceptor CCR5, whereas, as the disease progresses, HIV-1 selects coreceptor CXCR4 [5]–[21]. Given its sequence variability and structural flexibility, there must be an evolving physicochemical characteristic of the V3 loop that is responsible for switch in coreceptor selection during the progression of the disease. Previous studies have proposed that the main determinants for coreceptor selection are the V3 loop net charge [11]–[12], the presence of the glycosylation motif, spanning residues N6X7T8|S8X9, with glycan being attached at Asn6 [8], and the presence of one or more positive amino acids at positions 11/24/25 [9]. Both of the V3 loops examined in the present study have a highly conserved arginine at position 9 (92% conservation), which forms salt bridge-like interactions with a glutamic acid in position 25 (of the 11/24/25 rule) in 2B4C, or an aspartic acid at position 29 in 2QAD. We propose that as positive charge is increased when coreceptor selection switches from CCR5 to CXCR4, satisfying the 11/24/25 and net charge rules, Glu25 and Asp29 are amenable to replacement by a positively charged amino acid. This will not only break salt bridges, but will also introduce unfavorable Coulombic interaction with Arg9 (and other positively charged amino acids in the vicinity), with end-result the opening of the stem region of the V3 loop. It is likely that an open V3 loop favors interaction with CXCR4, whereas a thin V3 loop favors CCR5. The remainder tropism-determining parameter, the glycosylation motif, should also be included in this picture. The V3 loop of 2B4C is an X4-tropic sequence, as it is lacking the glycosylation motif because of an Asn6 to Gln6 mutation [4]. On the other hand, 2QAD has intact the glycosylation motif and a glycan attached to Asn6 in the original structure of the gp120-CD4-antibody complex [5]. Examination of the V3 loop crystallographic structures shows that 2B4C is an open structure, whereas 2QAD is a thin structure, at the stem region (Figure 1), implicating the glycan in stabilizing the β-strand of the stem, through either intra- or inter-molecular interactions. Based on the arguments presented above, we propose that upon replacing the negatively charged amino acids at positions 25/29 with positively charged, dissociation of the salt bridges occurs, followed by stem opening. Opening may be gradual with sequential replacements, perhaps starting with position 25 and following with 29 and/or other positions. Another mechanism for opening involves absence of the glycosylation motif and the interactions it carries. In either case, V3 loop stem opening should promote CXCR4 recognition.

In the case of the V3 loops studied here, their net charge is +3, but the spatial distributions of their electrostatic potentials vary during the MD trajectories, owed to conformational variations. Yet, our study shows some overlap in electrostatic potential similarities across the two trajectories, which we interpret as conserved electrostatic potential similarities that may be responsible for non-specific recognition of the coreceptor (either CCR5 or CXCR4), even in the presence of sequence differences and absence of specific structure. This hypothesis is valid within a two-step model of V3 loop – CCR5/CXCR4 association, with distinct steps of recognition and binding, and is in line with our previous studies [11]–[12], [20]. In this model recognition is non-specific and is driven entirely by long-range electrostatic interactions, and binding is specific and is driven by short-range pairwise polar and non-polar interactions and entropic effects.

Taking together our present data, and in view of previously published work, we propose that cooperativity between charged residue interactions and correlated motions is associated with a coreceptor binding specificity, whereas the property of bulk electrostatic potential is associated with coreceptor recognition. The described motions of the V3 loop suggest that it can acts as a hook to anchor the virus in the host cell, through V3 loop – coreceptor interaction, which has also been suggested by Kwong and coworker [4]. The same group has suggested that the binding of a CCR5-Nt peptide with two sulfated tyrosines at the V3 loop base, alters the V3 loop shape to accommodate the sulfated tyrosines, and induces a rigid β-hairpin conformation in the V3 loop [5]. All in all, our findings suggest that a future systematic and thorough MD study on a large number of V3 loops with diverse sequences, perhaps consensus sequences of HIV-1 subtypes, can provide insights into the different correlated motions and charged interactions of loops recognizing different coreceptors.

Many efforts, over several years, have been dedicated in the design of HIV drugs, and several post-entry drugs are approved by the FDA (Reviewed in [64]–[69]). However, no drug is suitable for all infections, as HIV is a moving target because of its sequence variability. Despite the fact that several entry inhibitors have been tested in clinical trials [64]–[65], [68], only one is currently approved by the FDA, a CCR5 antagonist [69]. Not surprisingly, resistance to this drug has been associated with mutations in the V3 loop, and it has been hypothesized that viral adaptation to CCR5 inhibition may result to selection of CXCR4 for viral entry [64]–[65], [69]. It has also been speculated that switch from CCR5 to CXCR4 due to viral adaptation, may result to acceleration of the disease [64]. The present study provides basic atomic resolution information on the stability and flexibility of the V3 loop, which can be used to address issues of drug resistance by HIV in future studies. Also, the present study provides useful insights into critical aspects of V3 loop peptide physicochemical characteristics, coupled to structure and dynamics, and offers molecular level understanding for future knowledge-based drug design.

## Supporting Information

Figure S1 **Root Mean Square Deviation** (RMSD) and **Root Mean Square Fluctuations.** (RMSF), in Å, for 2B4C (A) and 2QAD (B). The color code for RMSF (bottom) is: black, C_α_ atoms; red, backbone heavy atoms (N, C_α_, C); green, side chain heavy atoms; and blue, all heavy atoms per residue.
(TIF)

Figure S2 **Secondary Structure** **for (A) 2B4C, (B) 2QAD.** The colored code is: cyan, turn; yellow, extended conformations (extended β sheets); brown-green, isolated bridge; pink, alpha helix; blue, 3–10 helix; white, coil. The y-axis represent time, starting at 0 ns and ending at 100 ns. Secondary structure was determined using STRIDE implemented within VMD.
(TIF)

Figure S3 **Principal Component 2 Dynamic Cross-Correlation Maps for 2B4C (A) and 2QAD (B), using Cα atoms.** The color code for correlation or anti-correlation is shown at the right of each figure, with black being correlated, and yellow being anti-correlated. Axes denote the residue number in sequence. Bottom panels depict extreme structures observed during the principal components (shown in ribbon representation in blue and red) and the movements between structures (cyan).
(TIF)

Figure S4 **Charged Interactions within PC2 for 2B4C (A) and 2QAD (B).** Axes denote the residue number in sequence. Colors correspond to the extreme structures observed during the principal component 2 (bottom panels of Figure S3). Bottom panels show structures corresponding to local free energy minima of the FELs (Figure 7) for the second minima of 2B4C (left) and 2QAD (right). Negatively and positively charged residues involved in salt bridges are shown in red and blue, respectively, and disulfide bridge residues are shown in yellow. Salt bridges and β-bridges are marked with dashed lines. The backbone is shown in tube representation and the side chains are shown in stick representation. The base (residues 1–4, 31–35), stem (residues 5–10, 21–30) and tip (residues 11–20) regions are colored in cyan, black and purple color, respectively. The rest of the side chains are shown in thin pink licorice representation. Hydrogen atoms are omitted for clarity.
(TIF)

Figure S5 **Principal Component 3 Dynamic Cross-Correlation Maps** **for 2B4C (A) and 2QAD (B), using Cα atoms.** The color code for correlation or anti-correlation is shown at the right of each figure, with black being correlated, and yellow being anti-correlated. Axes denote the residue number in sequence. Bottom panels depict extreme structures observed during the principal components (shown in ribbon representation in blue and red) and the movements between structures (cyan).
(TIF)

Figure S6 **Charged Interactions within PC3** **for 2B4C (A) and 2QAD (B).** Axes denote the residue number in sequence. Colors correspond with the extreme structures observed during the principal component 3 (bottom panels of Figure S5). Bottom panels show structures corresponding to local free energy minima of the FELs (Figure 7) for the third minima of 2B4C (left) and 2QAD (right). Negatively and positively charged residues involved in salt bridges are shown in red and blue, respectively, and disulfide bridge residues are shown in yellow. Salt bridges and β-bridges are marked with dashed lines. The backbone is shown in tube representation and the side chains are shown in stick representation. The base (residues 1–4, 31–35), stem (residues 5–10, 21–30) and tip (residues 11–20) regions are colored in cyan, black and purple color, respectively. The rest of the side chains are shown in thin pink licorice representation. Hydrogen atoms are omitted for clarity.
(TIF)
