# Supplementary figures and images for: Insights into the Structure, Correlated Motions, and Electrostatic Properties of Two HIV-1 gp120 V3 Loops
Source: PLoS One. 2012 Nov 19;7(11):e49925. doi: 10.1371/journal.pone.0049925 (PMC3501474; doi:10.1371/journal.pone.0049925)

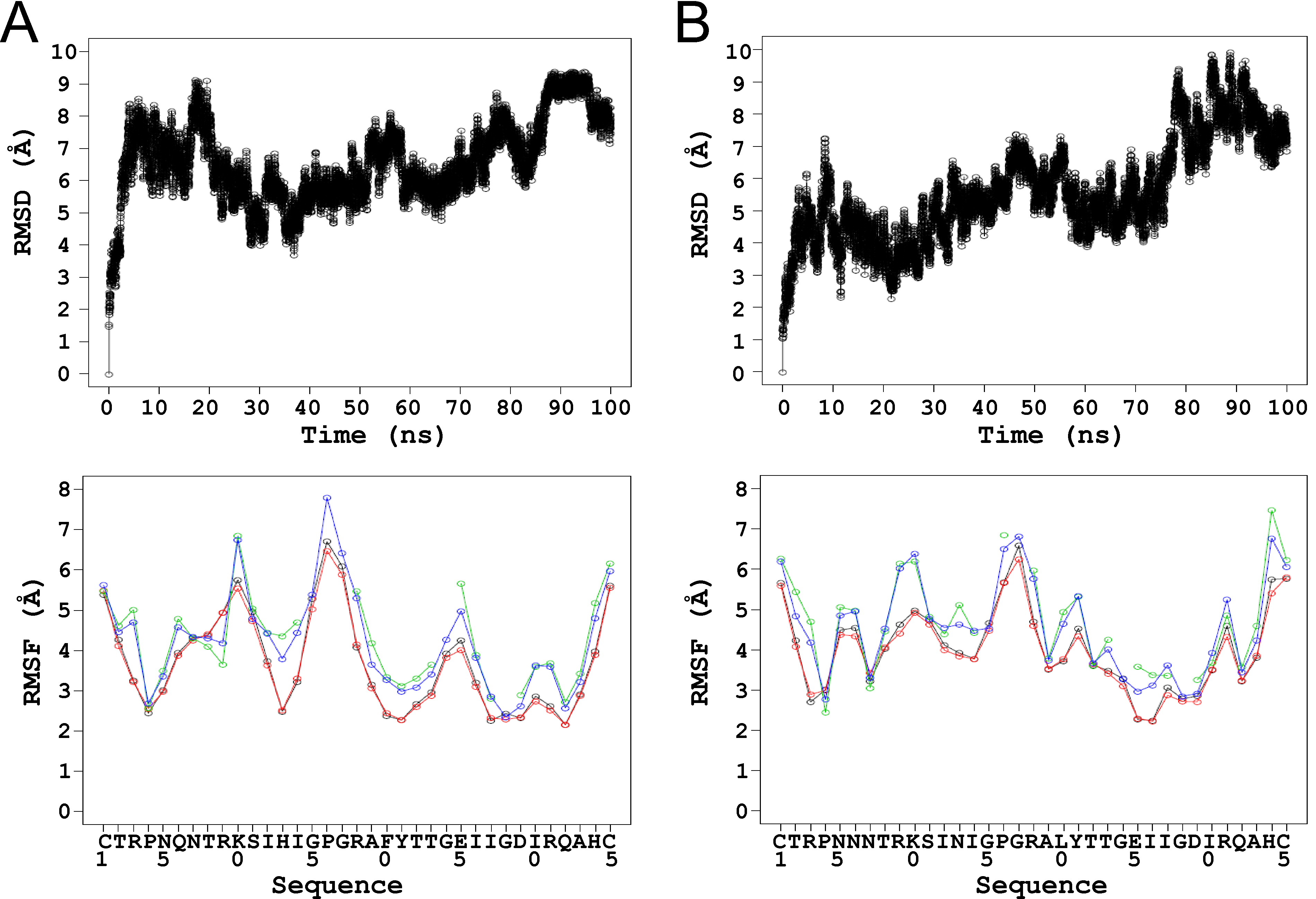

Supplement: Figure S1 — Root Mean Square Deviation (RMSD) and Root Mean Square Fluctuations. (RMSF), in Å, for 2B4C (A) and 2QAD (B). The color code for RMSF (bottom) is: black, Cα atoms; red, backbone heavy atoms (N, Cα, C); green, side chain heavy atoms; and blue, all heavy atoms per residue. (TIF) [file pone.0049925.s001.tif]

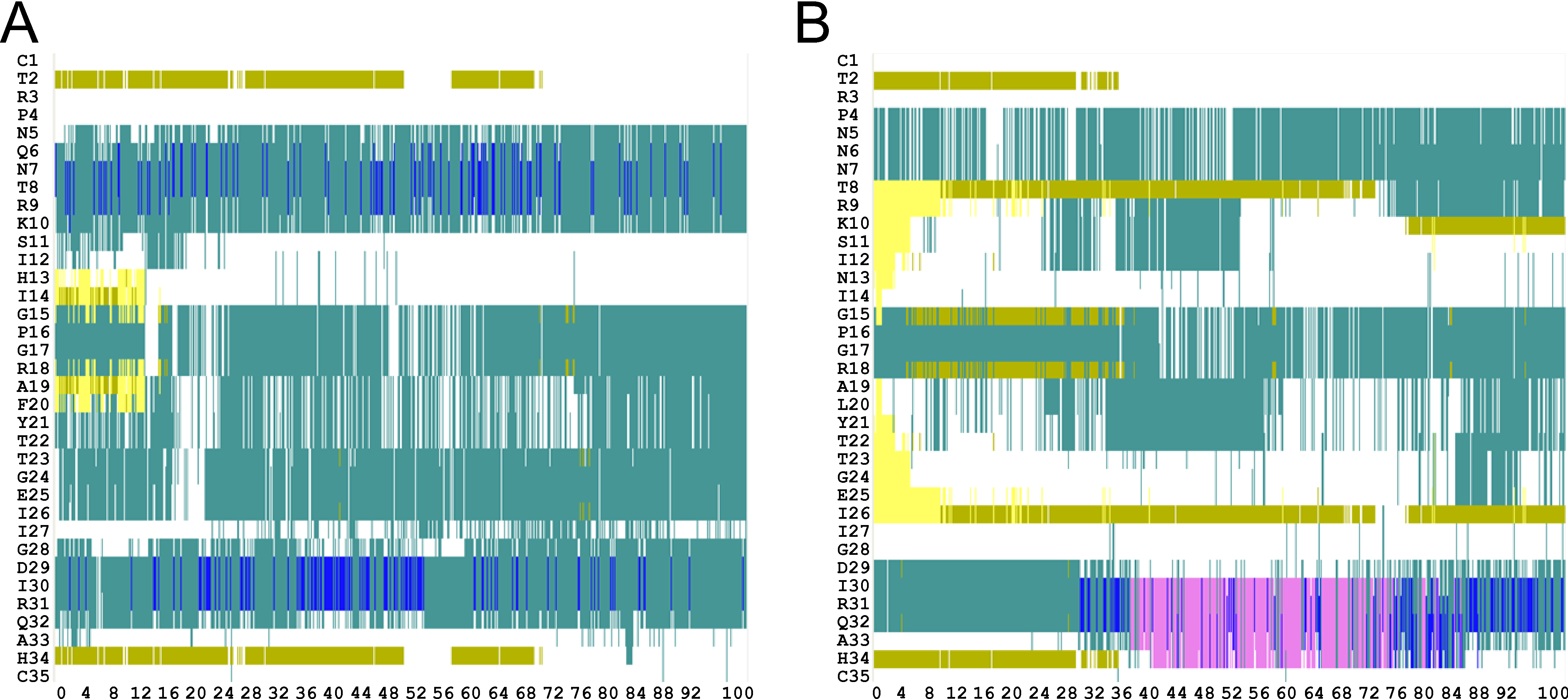

Supplement: Figure S2 — Secondary Structure for (A) 2B4C, (B) 2QAD. The colored code is: cyan, turn; yellow, extended conformations (extended β sheets); brown-green, isolated bridge; pink, alpha helix; blue, 3–10 helix; white, coil. The y-axis represent time, starting at 0 ns and ending at 100 ns. Secondary structure was determined using STRIDE implemented within VMD. (TIF) [file pone.0049925.s002.tif]

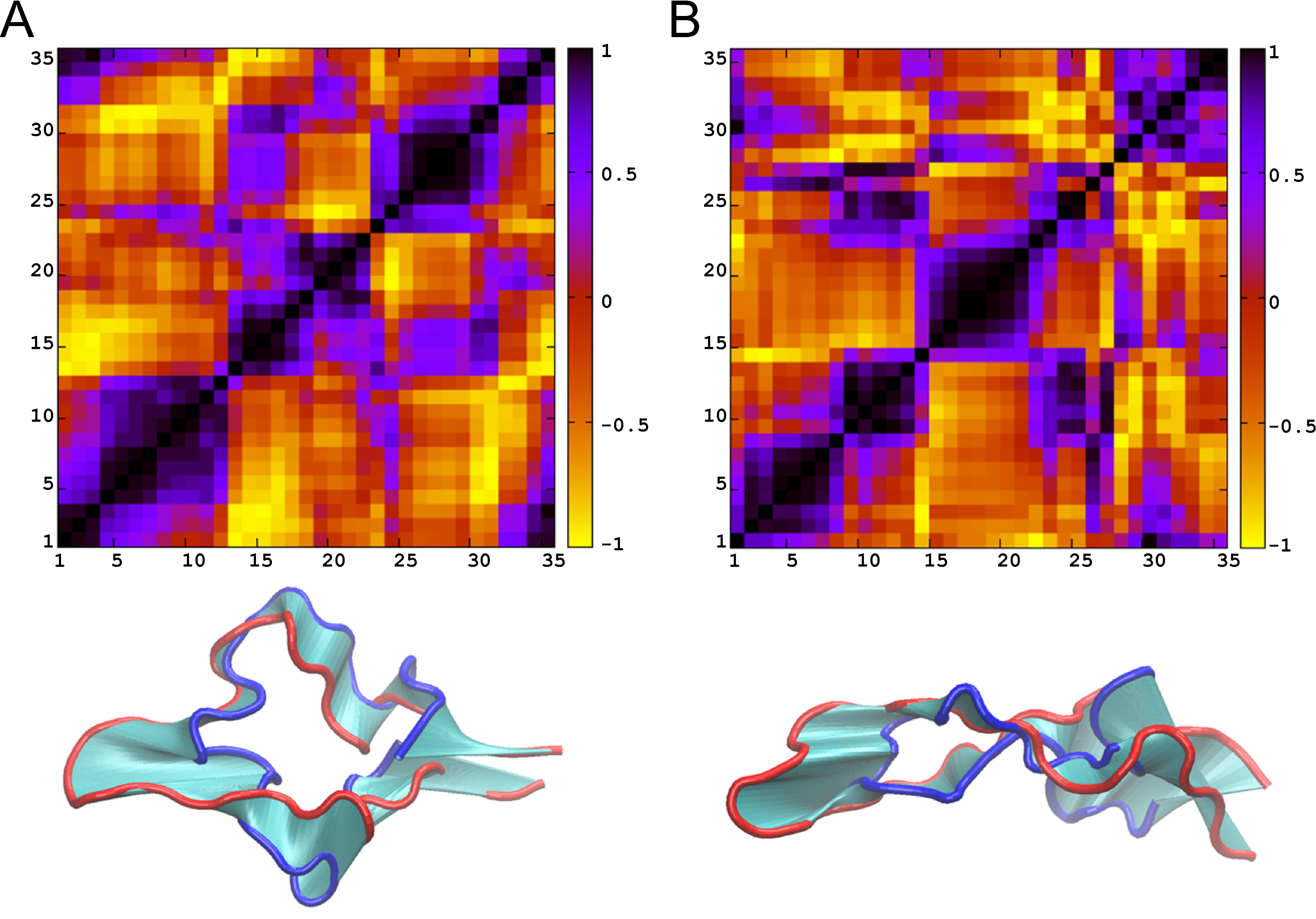

Supplement: Figure S3 — Principal Component 2 Dynamic Cross-Correlation Maps for 2B4C (A) and 2QAD (B), using Cα atoms. The color code for correlation or anti-correlation is shown at the right of each figure, with black being correlated, and yellow being anti-correlated. Axes denote the residue number in sequence. Bottom panels depict extreme structures observed during the principal components (shown in ribbon representation in blue and red) and the movements between structures (cyan). (TIF) [file pone.0049925.s003.tif]

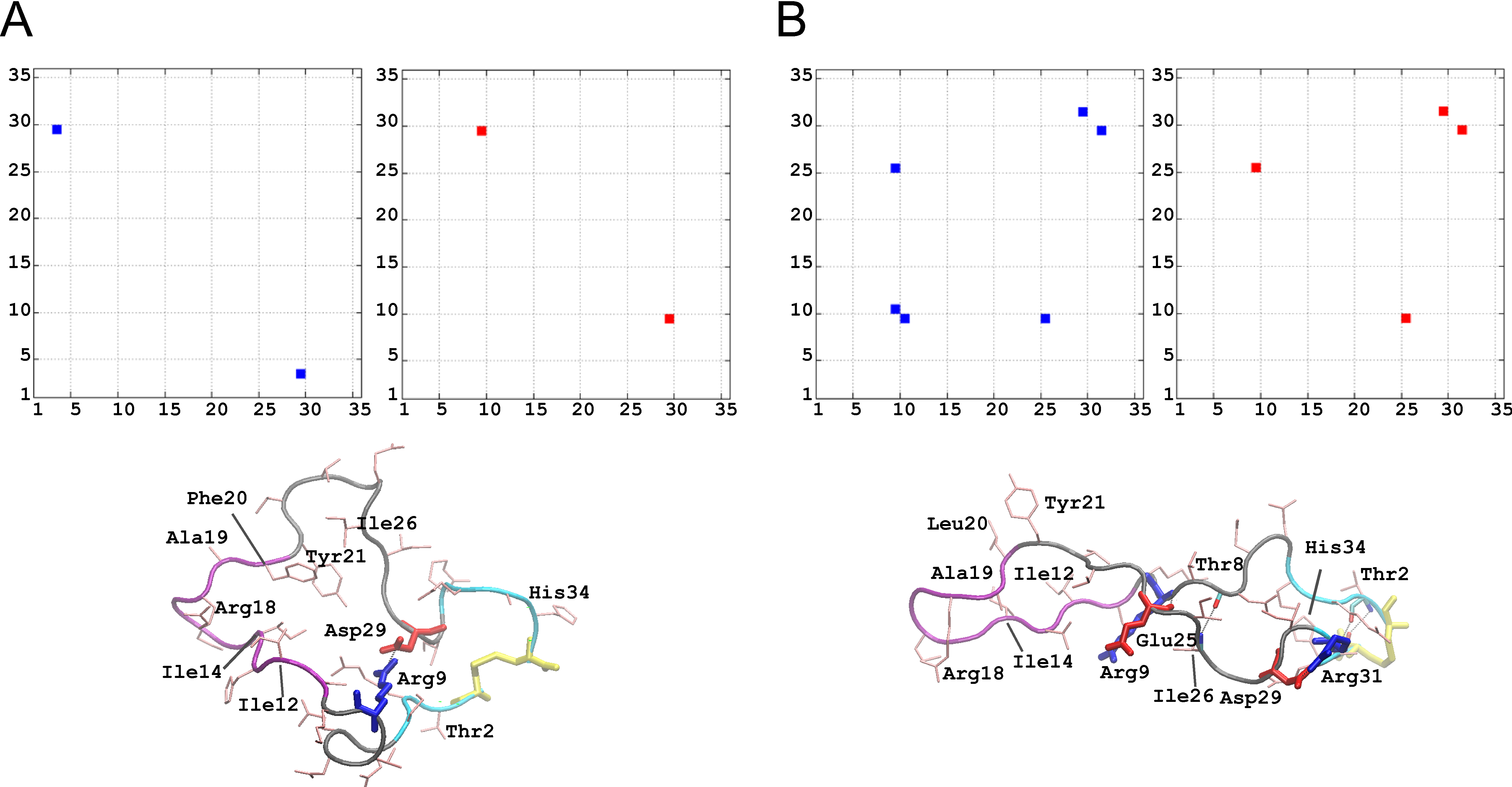

Supplement: Figure S4 — Charged Interactions within PC2 for 2B4C (A) and 2QAD (B). Axes denote the residue number in sequence. Colors correspond to the extreme structures observed during the principal component 2 (bottom panels of Figure S3). Bottom panels show structures corresponding to local free energy minima of the FELs (Figure 7) for the second minima of 2B4C (left) and 2QAD (right). Negatively and positively charged residues involved in salt bridges are shown in red and blue, respectively, and disulfide bridge residues are shown in yellow. Salt bridges and β-bridges are marked with dashed lines. The backbone is shown in tube representation and the side chains are shown in stick representation. The base (residues 1–4, 31–35), stem (residues 5–10, 21–30) and tip (residues 11–20) regions are colored in cyan, black and purple color, respectively. The rest of the side chains are shown in thin pink licorice representation. Hydrogen atoms are omitted for clarity. (TIF) [file pone.0049925.s004.tif]

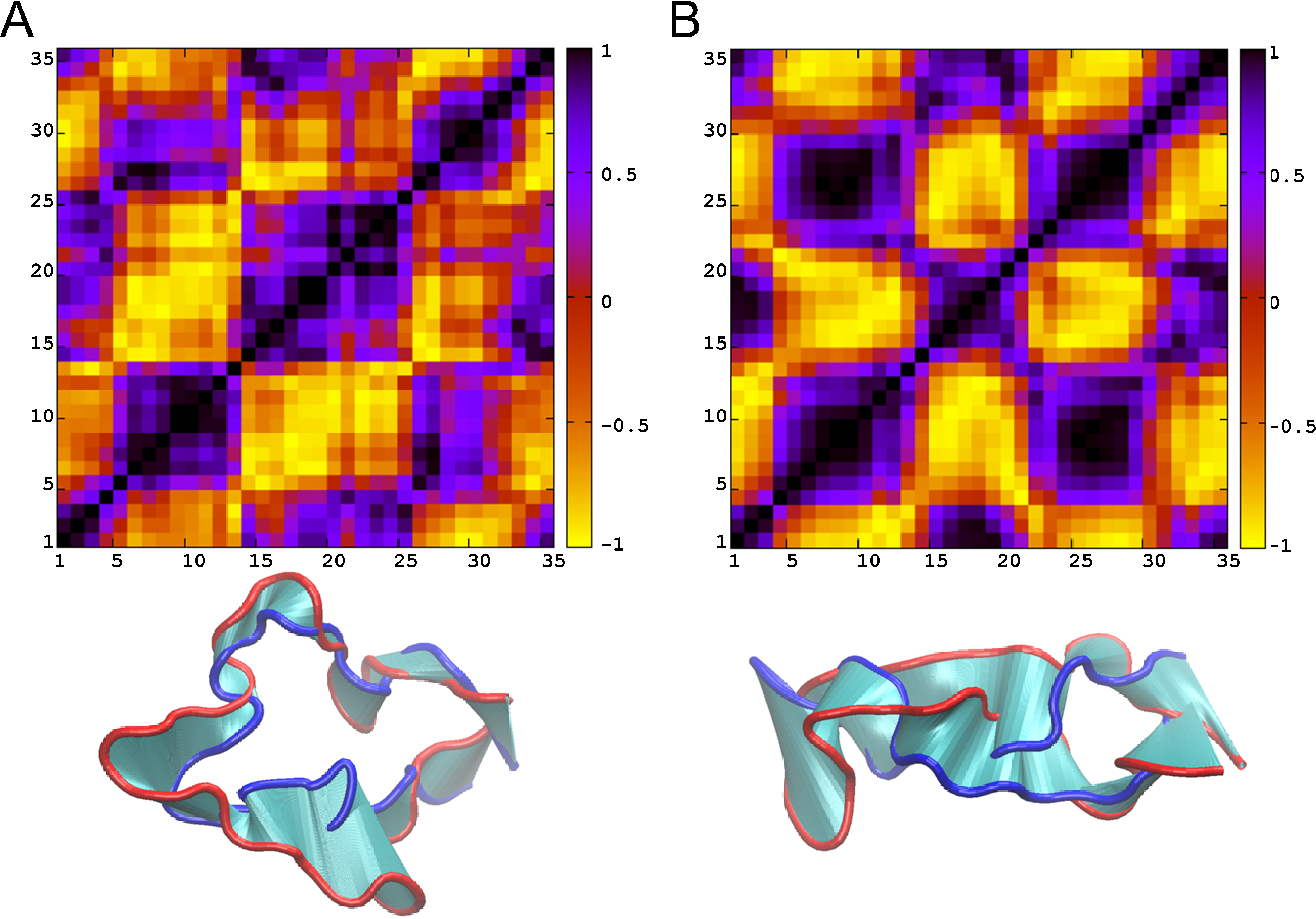

Supplement: Figure S5 — Principal Component 3 Dynamic Cross-Correlation Maps for 2B4C (A) and 2QAD (B), using Cα atoms. The color code for correlation or anti-correlation is shown at the right of each figure, with black being correlated, and yellow being anti-correlated. Axes denote the residue number in sequence. Bottom panels depict extreme structures observed during the principal components (shown in ribbon representation in blue and red) and the movements between structures (cyan). (TIF) [file pone.0049925.s005.tif]

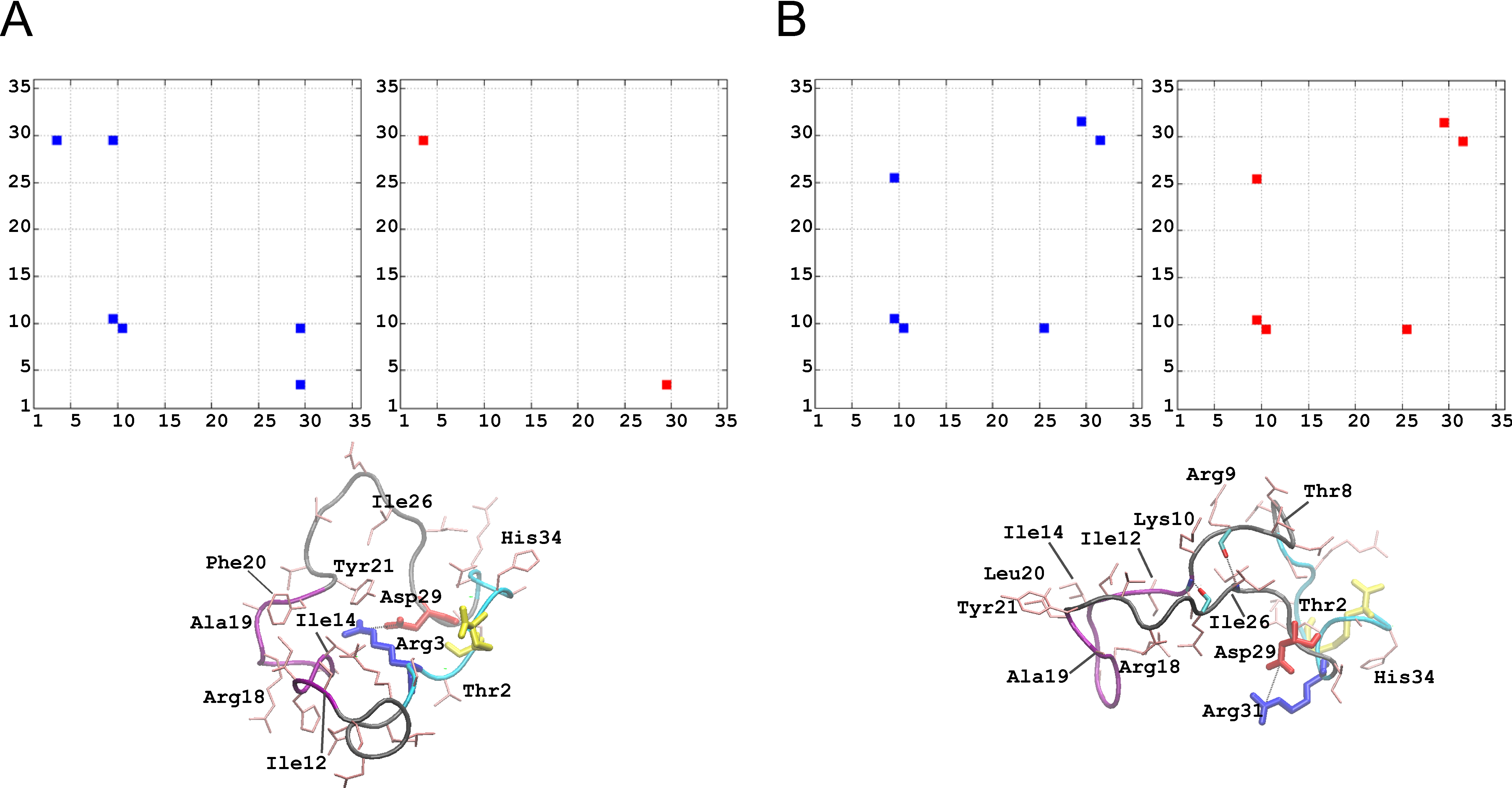

Supplement: Figure S6 — Charged Interactions within PC3 for 2B4C (A) and 2QAD (B). Axes denote the residue number in sequence. Colors correspond with the extreme structures observed during the principal component 3 (bottom panels of Figure S5). Bottom panels show structures corresponding to local free energy minima of the FELs (Figure 7) for the third minima of 2B4C (left) and 2QAD (right). Negatively and positively charged residues involved in salt bridges are shown in red and blue, respectively, and disulfide bridge residues are shown in yellow. Salt bridges and β-bridges are marked with dashed lines. The backbone is shown in tube representation and the side chains are shown in stick representation. The base (residues 1–4, 31–35), stem (residues 5–10, 21–30) and tip (residues 11–20) regions are colored in cyan, black and purple color, respectively. The rest of the side chains are shown in thin pink licorice representation. Hydrogen atoms are omitted for clarity. (TIF) [file pone.0049925.s006.tif]
